# Prognostic utility of plasma S100A12 levels to establish a novel scoring system for predicting mortality in maintenance hemodialysis patients: a two-year prospective observational study in Japan

**DOI:** 10.1186/1471-2369-14-16

**Published:** 2013-01-16

**Authors:** Yayoi Shiotsu, Yasukiyo Mori, Masato Nishimura, Tsuguru Hatta, Naoki Imada, Noboru Maki, Kumiko Iida, Noriyuki Iwamoto, Eiko Matsuoka, Keiichi Tamagaki, Atsushi Kosaki

**Affiliations:** 1 Division of Nephrology, Department of Medicine, Kyoto Prefectural University of Medicine, 465 Kajii-cho, Kawaramachi-Hirokoji, Kamigyo-ku, Kyoto, 602-8566, Japan; 2 Cardiovascular Division, Tojinkai Hospital, Kyoto, Japan; 3 Renal Division, Omihachiman Community Medical Center, Omihachiman, Japan; 4 Department of Urology, Nishijin Hospital, Kyoto, Japan; 5 Advanced Life Science Institute, Saitama, Japan; 6 Faculty of Nursing, Setsunan University, Hirakata, Japan

**Keywords:** Chronic kidney disease, Receptor for advanced glycation end products, Damage-associated molecular pattern molecules

## Abstract

**Background:**

S100A12 protein is an endogenous receptor ligand for advanced glycation end products. In this study, the plasma S100A12 level was assessed as an independent predictor of mortality, and its utility in clinical settings was examined.

**Methods:**

In a previous cross-sectional study, plasma S100A12 levels were measured in 550 maintenance hemodialysis patients to determine the association between S100A12 and the prevalence of cardiovascular diseases (CVD). In this prospective study, the risk of mortality within a two-year period was determined. An integer scoring system was developed to predict mortality on the basis of the plasma S100A12 levels.

**Results:**

Higher plasma S100A12 levels (≥18.79 ng/mL) were more closely associated with higher all-cause mortality than lower plasma S100A12 levels (<18.79 ng/mL; *P* = 0.001). Multivariate Cox proportional hazards analysis revealed higher plasma S100A12 levels [hazard ratio (HR), 2.267; 95% confidence interval (CI), 1.195–4.302; *P* = 0.012], age ≥65 years (HR, 1.961; 95%CI, 1.017–3.781; *P* = 0.044), serum albumin levels <3.5 g/dL (HR, 2.198; 95%CI, 1.218–3.968; *P* = 0.012), and history of CVD (HR, 2.068; 95%CI, 1.146–3.732; *P* = 0.016) to be independent predictors of two-year all-cause mortality. The integer score was derived by assigning points to these factors and determining total scores. The scoring system revealed trends across increasing scores for predicting the all-cause mortality [c-statistic = 0.730 (0.656–0.804)]. The resulting model demonstrated good discriminative power for distinguishing the validation population of 303 hemodialysis patients [c-statistic = 0.721 (0.627–0.815)].

**Conclusion:**

The results indicate that plasma S100A12 level is an independent predictor for two-year all-cause mortality. A simple integer scoring system was therefore established for predicting mortality on the basis of plasma S100A12 levels.

## Background

In 1960, introduction of the Scribner shunt allowed identification of continuous hemodialysis [1]. This advance greatly contributed to prolonged life in patients with kidney dysfunction. A growing number of patients throughout the world currently receive maintenance hemodialysis [2]. For example, in Japan alone, approximately 290,000 patients are regularly treated with hemodialysis [3].

Despite these and other successes in the past half century, use of dialysis in treatment of end-stage renal disease (ESRD) is still problematic in certain aspects. First, the efficiency of dialysis is still limited, and hemodialysis does not replace all normal kidney functions. Further, the body of a hemodialysis patient retains several molecules that provoke uremic toxicity, oxidative stress, and chronic inflammation [4]. Morbidity and mortality rates among hemodialysis patients consequently remain high despite considerable technical and scientific improvements in care and treatment of renal disease. The annual crude mortality rate of dialysis patients in Japan in 2009 was 9.6% according to the Renal Data Registry of the Japanese Society for Dialysis Therapy [3]. The mean life expectancy of the dialysis population in Japan was approximately 40%–60% of that of members of the general population of the same sex and age [5]. In addition, aggregate dialysis-associated costs have increased and have drained national healthcare budgets [2]. Therefore, each case of hemodialysis must be stratified by the prognostic risk of mortality, and an adjusted therapeutic plan must be designed for each hemodialysis patient.

The output of the axis of advanced glycation end products (AGE) and their receptor (RAGE) is enhanced in chronic kidney disease, particularly in ESRD [6,7]. AGE was originally considered the primary RAGE ligand, but additional RAGE ligands, including the high-mobility group box and S100 proteins, have also been identified [8]. S100A12 was the first member of the S100 family proven to initiate intracellular signaling by interacting with RAGE [9,10]. In ESRD patients, mean plasma S100A12 levels were found to be 2.3-fold higher than those in control subjects [11,12]. Thus, we hypothesized that the diagnostic value of plasma S100A12 level as a predictor of mortality may be superior to that of many conventional parameters because of its diverse pathophysiological activities via the AGE–RAGE axis such as chronic inflammation and tumorigenesis [13,14].

In the present study, the relationship between plasma S100A12 levels and all-cause mortality was assessed. A prospective observational study was conducted on patients undergoing maintenance hemodialysis. A simple prognostic score was developed for maintenance hemodialysis patients on the basis of their plasma S100A12 levels. Risk stratification according to the proposed scoring system may be useful in planning treatment in hemodialysis patients.

## Methods

### Study design

This was a prospective observational cohort study. All procedures were performed in accordance with the guidelines of the Helsinki Declaration on Human Experimentation and were approved by the Ethics Committees on Human Research of Kyoto Prefectural University of Medicine, Tojinkai Hospital, Omihachiman Community Medical Center, and Nishijin Hospital. All subjects provided informed consent. The study was performed in three independent dialysis centers in Japan and involved two years of patient follow-up. Demographic and medical data, including those on age, sex, smoking history, and comorbid conditions such as diabetes, were obtained from medical records.

### Subjects

Subjects were 550 hemodialysis patients from two centers (Tojinkai Hospital and Omihachiman Community Medical Center). These subjects were described in a previous cross-sectional study [11] and followed-up as a cohort (development population). Another cohort of 303 hemodialysis patients at an additional center (Nishijin Hospital) was followed-up as external validation population. Participants in this external population were enrolled in February 2009 according to the same protocol as that in the development population and observed prospectively until May 2011. All hemodialysis patients received conventional dialysis for 3–5 h three times per week with a standard bicarbonate dialysis solution, and all enrolled patients were clinically stable. None of the subjects showed clinical evidence of any malignant disease or overt infection. Basic patient characteristics, routine laboratory tests, and plasma S100A12 levels were determined at the beginning of the study. All-cause mortality was the outcome of interest. The date and cause of death were obtained by reviewing the hospital records. Cardiovascular mortality was defined as death attributable to myocardial ischemia and infarction, heart failure, cardiac arrest due to other or unknown causes, or cerebrovascular injuries [15]. Six patients in the validation population were excluded from the study because they moved to another dialysis center or were lost to follow-up.

### Laboratory methods

Blood samples were obtained via vascular access when dialysis was started on a midweek routine dialysis day. The plasma was immediately frozen and stored at −80°C until assay. The levels of urea nitrogen, serum creatinine, total cholesterol, albumin, hemoglobin, calcium, and phosphate were measured using standard clinical laboratory methods. White blood cell and platelet counts were also determined. The levels of high-sensitivity C-reactive protein (hs-CRP) were measured using an automatic analyzer (TBA-120FR; Toshiba, Tokyo, Japan).

### Quantitative ELISA for S100A12

A sandwich assay was performed using hCF128 and hCF113 monoclonal antibodies to quantify plasma S100A12 levels. S100A12 levels in the samples were determined by interpolation of absorbance values from a calibration curve. In our previous study, plasma S100A12 levels in 42 healthy subjects (29% men; mean age, 50.5 years) were determined; a median value of 9.4 ng/mL (6.8–12.9) was found [12].

### Statistical analyses

Results were expressed as means ± SD. Variables with a skewed distribution, including plasma S100A12 levels, were expressed as median and interquartile range (25th–75th percentiles). Group differences were analyzed using Student’s *t*-test, the Mann–Whitney U test, and the χ^2^ test for normally distributed, non-normally distributed, and discontinuous variables, respectively. Because our goal in this study was to arrive at a final prognostic score that could be easily interpreted and implemented in practice, all continuous variables were collapsed into the categorical variables. The normal range of plasma S100A12 levels in hemodialysis patients has not yet been standardized [11,16,17]. Therefore, the median value of the plasma S100A12 levels in the development population was used to dichotomize the data. Possible cutoff points for the other continuous variables for each laboratory test were determined according to their clinical importance using the results of previous studies and reference values [18-24]. The predictive value of the plasma S100A12 levels for two-year survival was estimated by the Kaplan–Meier analysis and evaluated using the log-rank test. The risk factors for all-cause mortality were first determined using a univariate Cox proportional hazards model, and variables with significant associations (*P* < 0.05) were then included in a multivariate Cox proportional hazards model in a stepwise procedure. The results of this multivariate analysis were then used to develop clinical prediction models.

Beta coefficients of variables were further assessed by the bootstrap method for internal validation and to reduce bias [25] and then rounded off. The prognostic risk scores of each individual patient were determined by assigning points for each factor and determining total scores. These total risk scores were then stratified into five categories for all-cause mortality according to the level of risk. The usefulness of the prediction score was subsequently evaluated by means of receiver operating characteristic curve analyses and c-statistics [25,26]. After the prediction score had been developed, it was verified using data from the external validation population comprising the 303 ESRD patients receiving maintenance hemodialysis at an additional independent center (Nishijin Hospital). All statistical analyses were performed using SPSS 20.0 for Windows (IBM Japan, Tokyo, Japan). A *P*-value <0.05 was considered statistically significant.

## Results

### Plasma S100A12 levels and all-cause mortality in the development population

**Figure 1:**
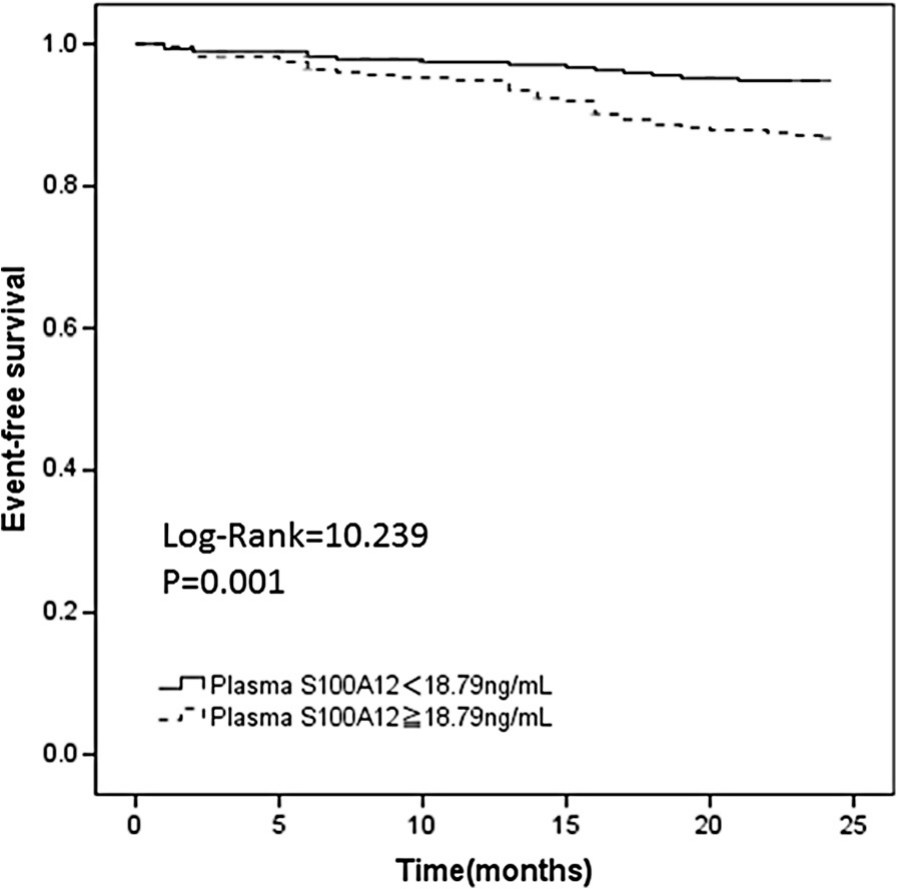
**Kaplan–Meier estimates of two-year survival by baseline plasma S100A12 levels.** The median value of plasma S100A12 levels of all participants at baseline were 18.79 ng/mL. The participants were divided into a lower S100A12 group (solid line) and a higher S100A12 group (dotted line) according to this median value (χ^2^ = 10.239, *P* = 0.001 by the log-rank test).

The development population included 550 ESRD patients who received maintenance hemodialysis in two independent dialysis centers belonging to our affiliated hospitals (65% men; mean age, 63.4 years; mean duration of hemodialysis, 9.7 years). Their underlying renal disorders included diabetic nephropathy (DN; 36.7%) and nonDN diseases such as chronic glomerulonephritis, hypertensive nephrosclerosis, polycystic kidney diseases, and others of unknown etiology (63.3%). A history of cardiovascular disease (CVD) was noted in 197 patients (35.8%). The median plasma S100A12 levels in these hemodialysis patients were 18.79 ng/mL (11.70–30.37). The clinical characteristics of the patients are listed in Table 1. During the median follow-up of 22.5 months, 50 patients (9%) died. Causes of death included cardiovascular events (n = 19); infection (n = 10); malignancy (n = 8); and other causes such as gastric intestinal bleeding, cachexia and trauma (n = 13). Significantly higher plasma S100A12 levels were observed in these patients than in those who survived [25.10 ng/mL (16.21–47.64) versus 17.93 ng/mL (11.16–29.28); *P* = 0.001; Table 1]. The Kaplan–Meier survival curves demonstrated that higher plasma S100A12 levels (≥18.79 ng/mL) were associated with higher all-cause mortality than lower S100A12 levels (<18.79 ng/mL; log-rank test, χ^2^ = 10.239, *P* = 0.001; Figure 1).

**Table 1:** Clinical characteristics and plasma S100A12 levels in the development population

	**All** (**n** = **550**)	**Died** (**n** = **50**)	**Survived** (**n** = **500**)	***P *value**^**a**^
Age (years)	63.4 ± 12.1^b^	69.9 ± 10.1	62.8 ± 12.1	<0.001**
Male/Female (n)	355/195	32/18	323/177	0.933
Current smoker/nonsmoker (n)	65/485	7/43	58/442	0.616
Duration of HD (years)	9.7 ± 8.4	9.7 ± 8.8	9.7 ± 8.3	0.969
Systolic BP (mmHg)	143.4 ± 26.0	143.4 ± 26.0	142.9 ± 22.0	0.878
Hemoglobin (g/dL)	10.3 ± 1.1	10.4 ± 1.2	10.3 ± 1.1	0.589
White blood cells (/mm^3^)	5729 ± 1816	5814 ± 1554	5720 ± 1841	0.728
Platelet (10^4^/μL)	16.8 ± 5.9	16.1 ± 6.0	16.8 ± 5.9	0.398
hs-CRP (mg/L)	1.0 (0.4–3.0)^c^	2.1 (0.8–6.3)	0.9 (0.4–2.7)	<0.001**
Creatinine (mg/dL)	10.5 ± 2.6	9.6 ± 2.8	10.6 ± 2.6	0.010*
Albumin (g/dL)	3.7 ± 0.4	3.5 ± 0.4	3.7 ± 0.4	<0.001**
Sodium (mEq/L)	139.5 ± 3.0	138.4 ± 3.0	139.6 ± 2.9	0.003**
Potassium (mEq/L)	4.6 ± 0.7	4.4 ± 0.8	4.6 ± 0.7	0.175
Adjusted calcium (mg/dL)	9.3 ± 0.6	9.2 ± 0.6	9.3 ± 0.6	0.380
Phosphate (mg/dL)	4.7 ± 1.1	4.5 ± 1.2	4.7 ± 1.1	0.283
Calcium (mg/dL) × Phosphate (mg/dL)	41.5 ± 10.5	39.0 ± 10.4	41.8 ± 10.5	0.074
Total cholesterol (mg/dL)	162.3 ± 45.8	158.4 ± 43.2	162.7 ± 46.1	0.534
DN/nonDN (n)	202/348	22/28	180/320	0.263
History of CVD/no history of CVD (n)	197/353	30/20	167/333	<0.001**
Plasma S100A12 levels (ng/mL)	18.79 (11.70–30.37)^c^	25.10 (16.21–47.64)	17.93 (11.16–29.28)	0.001**

^a^ Died versus survived. ^b^Means ± SD (all such values). ^c^Median value; 25%–75% value shown in parentheses (all such values). HD = hemodialysis, BP = blood pressure, hs-CRP = high-sensitivity C-reactive protein, DN = diabetic nephropathy, CVD = cardiovascular disease.

*P < 0.05, **P < 0.01.

The variables associated with all-cause mortality in the univariate Cox proportional hazards analysis are shown in Table 2. The following variables were significantly associated with all-cause mortality: high plasma S100A12 levels (≥18.79 ng/mL), age (≥65 years), hs-CRP levels (≥3 mg/L), serum albumin levels (<3.5 g/dL), and a history of CVD. Further, the multivariate Cox proportional hazards analysis incorporating these factors showed plasma S100A12 levels ≥18.79 ng/mL [hazard ratio (HR), 2.267; 95% confidence interval (CI), 1.195–4.302; *P* = 0.012] to be an independent predictor of all-cause mortality (Table 2). Other independent predictors were age ≥65 years (HR, 1.961; 95%CI, 1.017–3.781; *P* = 0.044), serum albumin levels <3.5 g/dL (HR, 2.198; 95%CI, 1.218–3.968; *P* = 0.012), and a history of CVD (HR, 2.068; 95%CI, 1.146–3.732; *P* = 0.016).

**Table 2:** Results of Cox’s proportional hazards analysis of clinical parameters and all-cause mortality

	**Univariate analysis**				**Multivariate analysis in a stepwise procedure**			
	**Beta coefficient**	**unadjusted HR**	**95**%**CI**	***P *value**	**Beta coefficient**	**adjusted HR**	**95**%**CI**	***P *value**
Age ≥65 years	0.972	2.643	1.425–4.900	0.002**	0.674	1.961	1.017–3.781	0.044*
Male	−0.003	0.997	0.560–1.776	0.992				
Current smoker	0.190	1.210	0.544–2.689	0.648				
Duration of HD ≥10 years	−0.068	0.934	0.520–1.677	0.819				
Systolic BP <110 mmHg	0.721	2.057	0.759–5.575	0.156				
110–140 mmHg	-	-	-	-				
≥140 mmHg	0.158	1.171	0.641–2.140	1.171				
Hemoglobin <10 g/dL	0.006	1.006	0.554–1.826	0.984				
10–11 g/dL	-	-	-	-				
≥11 g/dL	0.569	1.767	0.681–4.589	0.242				
White blood cells ≥10000/mm^3^	−0.112	0.894	0.123–6.472	0.911				
Platelet ≥35 × 10^4^/μL	0.330	0.719	0.175–2.959	0.648				
hs-CRP ≥3 mg/L	0.957	2.604	1.490–4.552	0.001**				
Albumin <3.5 g/dL	1.016	2.761	1.580–4.828	<0.001**	0.788	2.198	1.218–3.968	0.012*
Potassium ≥5.5 mEq/L	0.128	1.136	0.484–2.667	0.769				
Phosphate ≥6 mg/dL	−0.211	0.810	0.322–2.041	0.655				
Total Cholesterol ≥200 mg/dL	0.123	1.130	0.508–2.513	0.764				
DN	0.300	1.349	0.772–2.359	0.293				
History of CVD	1.045	2.844	1.615–5.009	<0.001**	0.727	2.068	1.146–3.732	0.016*
Plasma S100A12 levels ≥18.79 ng/mL	0.967	2.631	1.419–4.878	0.002**	0.919	2.267	1.195–4.302	0.012*

HD = hemodialysis, BP = blood pressure, hs-CRP = high-sensitivity C-reactive protein, DN = diabetic nephropathy, CVD = cardiovascular disease,

HR = hazard ratio, 95%CI = 95% confidence interval.

**P < 0.01, *P < 0.05.

### Development of the risk score

**Figure 2:**
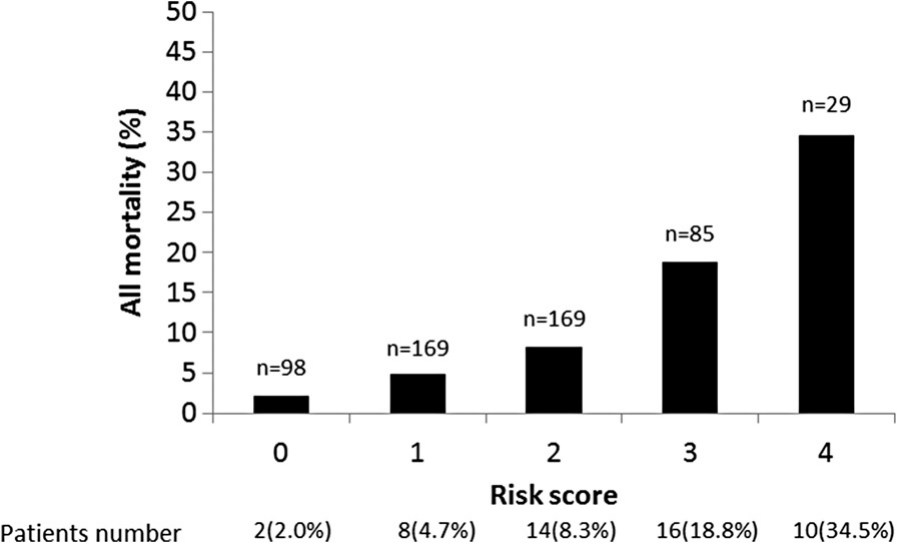
**Development of the risk score.** Increasing risk of the two-year all-cause mortality with increasing risk score is evident. n = total number of patients in each score.

Beta coefficients of the independent variables obtained by multivariate Cox proportional hazards analysis were assessed using 1000 bootstrap resamples. No significant difference between original beta and bootstrapped coefficients was observed (age, 0.674 vs. 0.713; albumin, 0.788 vs. 0.780; history of CVD, 0.727 vs. 0.730; plasma S100A12 levels, 0.919 vs. 0.935). One point (rounded off) was assigned to each of the four independent predictors of all-cause mortality (Table 3). The integer score obtained by assigning and totaling the points for each factor ranged from 0 to 4. All-cause mortality in hemodialysis patients by risk score assignment is depicted in Figure 2, with trends shown across increasing scores for predicting all-cause mortality. The two-year c-statistic of the development model was 0.730 (0.656–0.804).

**Table 3:** Integer risk score

**Predictor variable**	**Risk score value**
Age ≥65 years	1
Albumin <3.5 g/dL	1
History of CVD	1
Plasma S100A12 levels ≥ 18.79ng/mL	1
Total risk score	×/4

CVD = cardiovascular disease.

**Figure 3:**
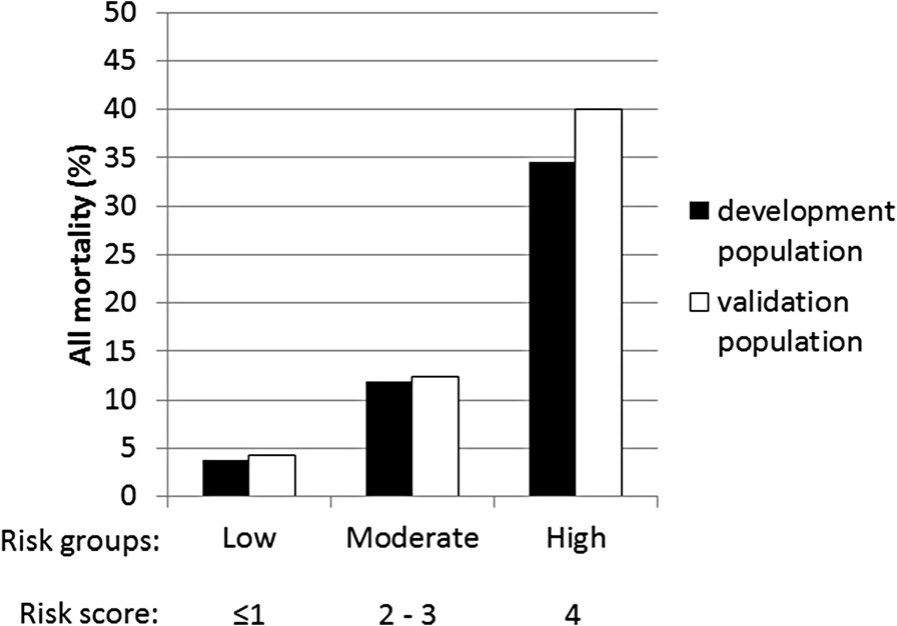
**External validation of the risk score.** The risk score derived from the development population also predicted the two-year all-cause mortality in the validation population. Solid bars = development population; open bar = validation population.

On the basis of the results for all-cause mortality as stratified by risk score, the 550 patients in the development population were further categorized into three groups as follows: low risk (*n* = 267; 48.5%), moderate risk (*n* = 254; 46.2%), and high risk (*n* = 29; 5.3%), corresponding to risk scores of ≤1, 2–3, and 4, respectively (Figure 3).

### External validation of the risk score

The validation population included 303 ESRD patients in whom maintenance hemodialysis was performed in another affiliated hospital. The clinical characteristics of these patients are listed in Table 4. In this validation population, the two-year all-cause mortality was 10.9% (n = 33). Causes of death included cardiovascular events (n = 18); infection (n = 9); malignancy (n = 4); and other causes such as cirrhosis and cachexia (n = 2). The values for the all-cause mortality in the validation population were close to those in the development population in each of the three risk groups (Figure 3). The developed model demonstrated good discriminative power in the validation population [c-statistic = 0.721 (0.627–0.815)].

**Table 4:** Clinical characteristics and plasma S100A12 levels in the validation population

	**All** (**n** = **303**)	**Died** (**n** = **33**)^**a**^	**Survived** (**n** = **264**)^**a**^	***P *value**^**b**^
Age (years)	69.4 ± 11.7^**c**^	74.9 ± 10.5	68.7 ± 11.4	0.003**
Male/Female (n)	195/108	27/6	165/99	0.029*
Current smoker/nonsmoker (n)	51/252	5/28	46/218	0.744
Duration of HD (years)	7.4 ± 7.2	6.6 ± 4.4	7.6 ± 7.5	0.254
Systolic BP (mmHg)	148.3 ± 23.0	146.2 ± 26.7	148.5 ± 22.6	0.594
Hemoglobin (g/dL)	10.4 ± 1.2	9.9 ± 1.4	10.5 ± 1.2	0.007**
White blood cells (/mm^3^)	5438 ± 1579	5867 ± 1707	5393 ± 1557	0.104
Platelet (10^4^/μL)	17.7 ± 5.8	16.8 ± 5.8	17.7 ± 5.8	0.380
hs-CRP (mg/L)	0.9 (0.4–3.4) ^d^	1.4 (0.6–7.8)	0.9 (0.4–3.0)	0.013*
Creatinine (mg/dL)	9.0 ± 2.8	7.4 ± 2.6	9.3 ± 2.7	<0.001**
Albumin (g/dL)	3.6 ± 0.4	3.3 ± 0.5	3.6 ± 0.3	<0.001**
Sodium (mEq/L)	139.6 ± 2.8	138.8 ± 3.5	139.7 ± 2.6	0.172
Potassium (mEq/L)	4.9 ± 0.8	4.7 ± 0.8	4.9 ± 0.8	0.076
Adjusted calcium (mg/dL)	9.1 ± 0.7	9.2 ± 0.6	9.1 ± 0.7	0.404
Phosphate (mg/dL)	4.9 ± 1.2	4.6 ± 1.4	5.0 ± 1.2	0.162
Calcium (mg/dL) × Phosphate (mg/dL)	42.7 ± 11.5	39.5 ± 11.9	43.3 ± 11.5	0.078
Total cholesterol (mg/dL)	146.6 ± 34.3	142.6 ± 42.9	147.3 ± 32.7	0.453
DN/nonDN (n)	132/171	21/12	110/154	0.017*
History of CVD/no history of CVD (n)	86/217	15/18	69/195	0.020*
Plasma S100A12 levels (ng/mL)	20.39 (12.65–37.98)^d^	28.60 (14.18–43.80)	19.45 (12.54–35.46)	0.035*

^a^Six patients were excluded from the final evaluation because they moved to another dialysis center or were lost to follow-up. ^b^Died versus survived. ^c^Means ± SD (all such values). ^d^Median value; 25%–75% value shown in parentheses (all such values). HD = hemodialysis, BP = blood pressure, hs-CRP = high-sensitivity C-reactive protein, DN = diabetic nephropathy, CVD = cardiovascular disease.

*P < 0.05, **P < 0.01.

## Discussion

This study followed-up 550 patients on maintenance hemodialysis after measurement of their plasma S100A12 levels. The main findings of the present study are as follows. A higher plasma S100A12 level (≥18.79 ng/mL) in hemodialysis patients was a significant predictor of the two-year all-cause mortality. In addition to the plasma S100A12 levels, age ≥65 years, serum albumin levels <3.5 g/dL, and a history of CVD were independent predictors of the two-year all-cause mortality. The predicting integer score developed using these four factors provided statistically significant information. Finally, external validation in a cohort of 303 hemodialysis patients at an independent center demonstrated that the simple integer score proposed here provided good discriminative power, and therefore serves as a useful predictive model of mortality in hemodialysis patients.

Prolonged subclinical inflammation causes release of intracellular molecules that alert the immune system to danger. Recent studies reported that these endogenous damage-associated molecular pattern (DAMP) molecules or alarmins, which are released from necrotic cells and activated leucocytes, play a crucial role in the inflammatory response [27]. Examples of putative DAMPs include endogenous RAGE ligands such as high-mobility group box 1 and S100 proteins (S100A8/A9 and S100A12), interleukins (IL) such as IL-1α, heat-shock proteins, and nucleosome [27]. The S100A12 expression is high in inflammatory diseases such as rheumatoid arthritis, Crohn’s disease, Kawasaki disease, and atherosclerosis [28-31]. Atherosclerosis, which causes CVD, is also characterized by the presence of subclinical chronic inflammation [32]. Furthermore, abnormal persistence of DAMPs in chronic inflammation and in tumor microenvironments is associated with carcinogenesis [14]. Signals that activate RAGE, which is also regarded as a prototypic DAMP receptor, may trigger a positive regulatory loop that maintains the inflammatory microenvironment required for the promotion of tumor development [13]. Thus, the interaction of S100A12 with RAGE may be involved in diverse processes such as inflammatory-induced atherosclerosis, defense against infection, and tumorigenesis. These factors contribute to the all-cause mortality in hemodialysis patients; thus, measurement of S100A12 levels may provide a sensitive discriminative predictor.

Two observational studies have been conducted examining the relationship between mortality and S100A12 in hemodialysis patients. Nakashima *et al*. [16] performed an *ad hoc* analysis of 184 prevalent hemodialysis patients in Sweden. They showed that the S100A12 level was an independent predictor of the all-cause and CVD-related mortality. Adjustment for plasma IL-6 levels as an inflammatory marker significantly attenuated the association with the all-cause mortality but not with respect to cardiovascular mortality. Because plasma IL-6 levels were not measured in this larger prospective study, a direct comparison with the results of the Swedish study was not possible. The low mortality rate in the current study (9%) did not allow statistical evaluation of the function of each cause of death, probably because of differences in the circumstances between dialysis facilities. Very recently, a prospective study of 261 Czech hemodialysis patients by Kalousova *et al*. [17] found a relationship between S100A12 levels and infection-related mortality. In contrast to the present study and the Swedish study, they found no difference between baseline S100A12 levels in the hemodialysis patients and those of healthy controls. In addition, interstitial nephritis and polycystic kidney disease were present in 26.8% and 14.6% of patients in that study, respectively; these values differed significantly from those in our subjects. Therefore, direct comparison between their study results and those of the current study was difficult. However, the relationship between mortality due to infection and S100A12 should be considered.

The risk scoring system developed in this study, which included the plasma S100A12 levels, was useful in predicting the two-year all-cause mortality. A score of 0–4 was calculated using data for age, albumin levels, history of CVD, and S100A12 levels derived from analysis of 550 participants. This score successfully predicted mortality even in the validation population. Furthermore, this simple model, which requires no extensive computation, can be used in clinical settings for rapid evaluation of the patient status. Considering the risk stratification and planning involved in the management of hemodialysis patients, this score system may provide useful and meaningful information to practitioners. Because the population of hemodialysis patients is increasing and because of economic and medical staff limitations, intensive treatment of all the patients who received maintenance hemodialysis is not possible [33,34]. For hemodialysis patients at high risk according to our proposed scoring system, a strong intention to treat, such as more careful observation of patient status; and shorter intervals between imaging studies to screen for CVD, malignancy, and infectious disease are recommended. In this context, the proposed scoring system may be a useful risk stratification tool in clinical settings. Prospective verification of the clinical usefulness of this system in our affiliated clinics is planned in future.

Some limitations of the present study must be acknowledged. First, prevalent but not incident dialysis patients were selected for inclusion. Second, although the sample size analyzed here was larger than that in previous studies performed in Sweden and Czech Republic [16,17] on the relationship between S100A12 levels and mortality in hemodialysis patients, the total number of deaths in this Japanese study was small. However, one report suggested that clinical prognosis is better in Japanese hemodialysis patients compared with those in other countries [35]. Third, other possible risk factors for mortality in ESRD patients, such as vascular calcification, nutritional state, subjective comorbid score, levels of candidate markers (brain natriuretic peptide, fetuin-A, and IL-6), dialysis adequacy, and residual renal function may be important, but were not measured here. Finally, the diagnostic assays currently used for measurement of S100A12 levels are useful for research purposes only. To date, no commercially available routine tests have become available for determining S100A12 levels in clinical studies. In addition, normal and reference plasma values are still under debate.

## Conclusion

In conclusion, in the present study of 550 maintenance hemodialysis patients, the plasma S100A12 level was verified as an independent predictor of two-year mortality. The simple integer score was shown to be successful in predicting mortality using four significant variables, including plasma S100A12 levels. In addition to studies verifying the usefulness of the proposed scoring system, interventional studies involving S100A12 are further planned to clarify the relationship between plasma S100A12 levels and hemodialysis mortality.

## Abbreviations

AGE: Advanced glycation end products; CI: Confidence interval; CVD: Cardiovascular diseases; DAMP: Damage-associated molecular pattern; DN: Diabetic nephropathy; ESRD: End-stage renal disease; HR: Hazard ratio; Hs-CRP: High-sensitivity C-reactive protein; IL: Interleukins; RAGE: Receptor for advanced glycation end products.

## Competing interests

The authors declare that they have no competing interests.

## Authors’ contributions

YS collected, analyzed, and interpreted the data and drafted the manuscript. YM conceptualized the study and its objective, supervised the conducting of the study, and also drafted the manuscript. MN, TH, NI, and NI (Iwamoto) extracted the data and provided the clinical information. NM and KI measured and analyzed the plasma S100A12 levels. EM and KT helped to interpret the data and draft the manuscript. AK participated in the design and supervised the conduct of the study with YM. All authors read and approved the final manuscript.

## Pre-publication history

The pre-publication history for this paper can be accessed here:

http://www.biomedcentral.com/1471-2369/14/16/prepub
